# Film Growth Rates and Activation Energies for Core-Shell Nanoparticles Derived from a CVD Based Aerosol Process

**DOI:** 10.3390/ma8030966

**Published:** 2015-03-06

**Authors:** Frederik Weis, Martin Seipenbusch, Gerhard Kasper

**Affiliations:** 1 Institute for Mechanical Process Engineering and Applied Mechanics, Karlsruhe Institute of Technology (KIT), Strasse am Forum 8, 76131 Karlsruhe, Germany; E-Mail: gerhard.kasper@kit.edu; 2 Institute of Chemical Process Engineering, University of Stuttgart, Böblinger Strasse 78, 70199 Stuttgart, Germany; E-Mail: martin.seipenbusch@icvt.uni-stuttgart.de

**Keywords:** core-shell, chemical vapor deposition (CVD), nanoparticles, coating, molybdenum oxide, bismuth oxide, activation energy, aerosol

## Abstract

Silica core-shell nanoparticles of about 60–120 nm with a closed outer layer of bismuth or molybdenum oxide of 1–10 nm were synthesized by an integrated chemical vapor synthesis/chemical vapor deposition process at atmospheric pressure. Film growth rates and activation energies were derived from transmission electron microscopy (TEM) images for a deposition process based on molybdenum hexacarbonyl and triphenyl bismuth as respective coating precursors. Respective activation energies of 123 ± 10 and 155 ± 10 kJ/mol are in good agreement with the literature and support a deposition mechanism based on surface-induced removal of the precursor ligands. Clean substrate surfaces are thus prerequisite for conformal coatings. Integrated aerosol processes are solvent-free and intrinsically clean. In contrast, commercial silica substrate particles were found to suffer from organic residues which hinder shell formation, and require an additional calcination step to clean the surface prior to coating. Dual layer core-shell structures with molybdenum oxide on bismuth oxide were synthesized with two coating reactors in series and showed similar film growth rates.

## 1. Introduction

Nanoparticles with core-shell structures have wide applications ranging from catalysis to medical applications and optoelectronic devices. As an alternative to conventional multistep liquid phase synthesis routes, several gas phase processes have been developed and proven to offer continuous, solvent-free and scalable synthesis methods [1,2,3]. Such an integrated chemical vapor synthesis (CVS)/chemical vapor deposition (CVD) process was used to produce well defined core-shell structures with a silica core and an oxide outer layer. A key feature of this approach is that spherical core particles are prepared continuously by decomposition of tetra-ethyl-ortho-silicate (TEOS), and then transferred immediately to a CVD coating step with molybdenum and/or bismuth oxide. These coating materials were selected, since they have potential as supported thin film catalysts for the selective oxidation of hydrocarbons [4,5,6,7]. MoO_3_ and Bi_2_O_3_ were furthermore investigated as electrochromic materials in “smart” windows and display devices [8], as an ion conducting electrolyte material in solid oxide fuel cells, as gas sensors [9] and nanostructured photocatalysts for the degradation of organic compounds [10]. Prerequisite for a workable coating process via CVS/CVD is the availability of suitable precursors with good thermal stability combined with clean evaporation or sublimation. Commercially available molybdenum hexacarbonyl and triphenyl bismuth were found to meet these requirements, are stable upon storage and handling and were therefore selected. CVD of MoO_3_ and Bi_2_O_3_ on planar substrates with these precursors has been reported [11,12,13,14,15,16]. However, little can be found with regard to the coating of micron sized or even submicron particles with such materials. To the best of our knowledge, only fluidized bed techniques have been used to date for the coating of micronsized particles with molybdenum oxide [17,18,19] or bismuth oxide [20] with focus on the subsequent catalytic application, not on the coating process itself.

The aim of this work was thus to identify the optimal process parameters for the synthesis of such core-shell structures in an aerosol process, and to derive the deposition kinetics for Mo and Bi required for future optimization and scale-up. Furthermore, comparisons of coating results were made with coatings on commercial carrier particle materials.

## 2. Results and Discussion

**Figure 1:**
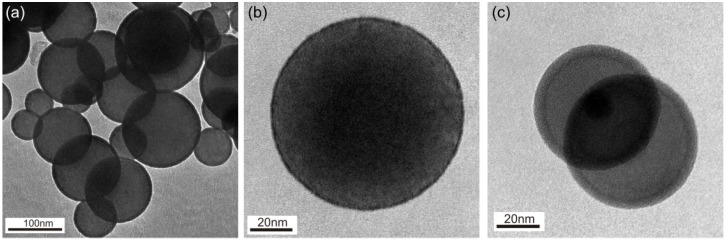
TEM images of core-shell structures synthesized by a (CVS)/(CVD) at atmospheric pressure, comprising a silica core and outer layers of (**a**) bismuth oxide at 390 °C; (**b**) bismuth oxide at 370 °C and (**c**) molybdenum oxide at 130 °C.

For both material systems perfect core-shell structures could be synthesized as shown in Figure 1.

### 2.1. Molybdenum Oxide Coatings

**Figure 2:**
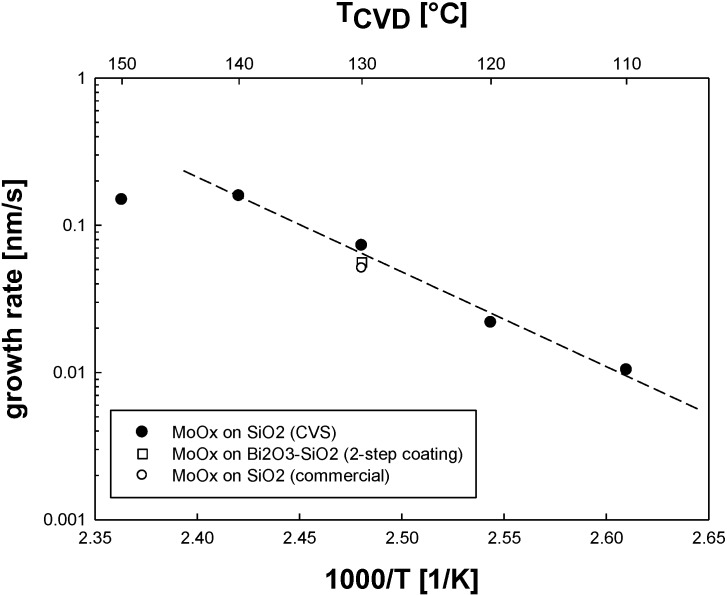
Arrhenius plot for CVD based growth rates of molybdenum oxide films on silica particles generated freshly by CVS (black dots), silica particles generated by CVS and coated with an additional layer of bismuth oxide (open square), and commercially available silica particles (open dot). The dashed line fit was used to derive the activation energy.

Growth rates for molybdenum oxide films on silica particles were derived from the analysis of a series of TEM images at a residence time of 50 s in the coating reactor. (Deriving the thickness of individual layers based on a recently described and very sensitive online-technique [21] is also possible, but requires exact density values for each layer. Unfortunately different values are reported in the literature for molybdenum oxide, either as MoO_3_ or MoO_2_, and one cannot be sure of some interdiffusion for double layers). No film growth was detected below 110 °C; above 140 °C no further increase of the film growth rate was observed due to the onset of competing homogeneous gas phase decomposition of the precursor resulting in the formation of fractal agglomerates of molybdenum oxide. An Arrhenius plot (Figure 2) with an exponential fit in the temperature range 110–140 °C gives an activation energy of 123 ± 5 kJ/mol for the film growth rate. This value matches the activation energy for the first dissociative CO loss as determined by Loh *et al.* [22] by temperature programmed decomposition of molybdenum hexacarbonyl on silica substrates. As expected, it is also lower than the activation energy required for autocatalytic homogeneous gas phase decomposition, which is reported in the range of 150–164 kJ/mol [23,24].

**Figure A1:**
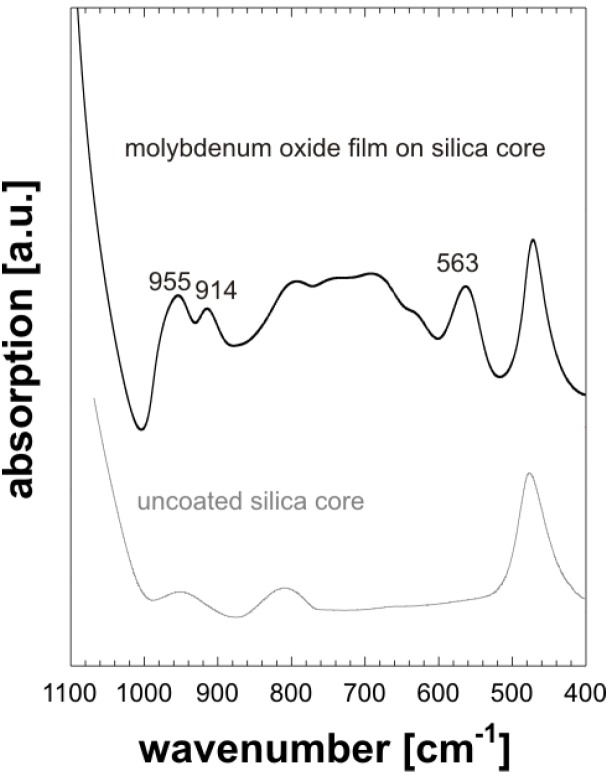
FTIR spectra of the silica particles before and after coating with molybdenum oxide by combined CVS/CVD. The additional peaks in the coated sample at 955, 914 and 563 cm^−1^ can be assigned to terminal double-bond Mo=O vibrations in surface molybdates [32], Mo–O–Si linkage vibrations [33] and single bond Mo–O vibration in octahedral MoO_6_ units [34], respectively. The broad band in the range of 600–900 cm^−1^ is due to overlapping Mo–O single bond and Mo–O–Mo vibrations of Mo–Ions in tetrahedral and octahedral coordination and typically observed in nonstoichiometric molybdenum oxides [35].

Prerequisite for a successful coating is the chemisorption and subsequent decomposition of the precursor on the silica particle surface. For the molybdenum hexacarbonyl precursor this occurs via adsorption on surface hydroxyl groups and an exchange of one CO ligand, followed by successive complete decarbonylation in a single step reaction with the activation energy of about 122 kJ/mol given by Loh *et al.* [22]. However, once the first monolayer coverage is reached in our process, further film growth continues via adsorption of the precursor molecules on molybdenum oxide layers and thus probably with a different activation energy. Unfortunately, there is no literature for the adsorption and decomposition of molybdenum hexacarbonyl on molybdenum oxide. Nonetheless, our data indicate that the activation energy does not change significantly with film thickness or compared to the plain silica substrate. According to Loh *et al.* [22] the activation energy for the decarbonylation is a function of the field strength of the substrate cation, namely the Si^4+^ ion in their studies, attached to the surface hydroxyl group, which has to be exchanged. The field strength is defined as the cation charge divided by the ionic radius (0.42 Angstrom for Si^4+^ in SiO_2_), yielding a field strength of 9.5 Å^−1^ for silica. Applying the same calculations to molybdenum-(VI)-oxide with an ionic radius of 0.62 Angstrom for Mo^6+^ as given by Pauling [25] results in a field strength of 9.6 Å^−1^, which is very close to silica and thus supports the assumption that the film growth rate is limited by the decarbonylation step with an activation energy of about 123 ± 5 kJ/mol. However, some uncertainties arise regarding the state of molybdenum ions, since for the as-deposited molybdenum oxide film no distinct crystalline peaks could be observed in the WAXS (wide angle X-ray) spectrum, most likely due to the very thin film and/or its partially amorphous state. Moreover, the Fourier transform infrared (FTIR) spectrum of the coated particles (see Figure A1) shows features of molybdenum blue oxide, which is an unordered, complex molybdenum phase and typically contains a mixture of Mo^5+^/Mo^6+^-Ions. Therefore, calculations were also done for Mo^5+^ with an effective ionic radius of 0.46–0.61 Angstrom as given by Shannon [26]. The range of ionic radii includes uncertainties regarding the coordination number of the Mo^5+^ ions, which might be less coordinated on the surface and could either be 4 or 6. Nonetheless the calculated field strength of 8.2–10.9 Å^−1^ results in an activation energy of 117–124 kJ/mol according to the data given by Loh *et al.* and is thus in good agreement with our experimental value of about 123 ± 5 kJ/mol.

**Figure 3:**
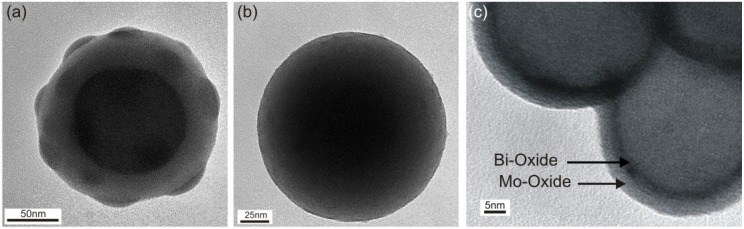
TEM images of (**a**) commercial silica particles (AngstromSphere™) used as-received and coated with molybdenum oxide, where residues on the particle surface hinder conformal coating; (**b**) the same commercial silica particles calcinated prior to coating to remove residues and then coated with molybdenum oxide; (**c**) CVS generated silica particles coated successively with bismuth oxide and molybdenum oxide.

**Figure A2:**
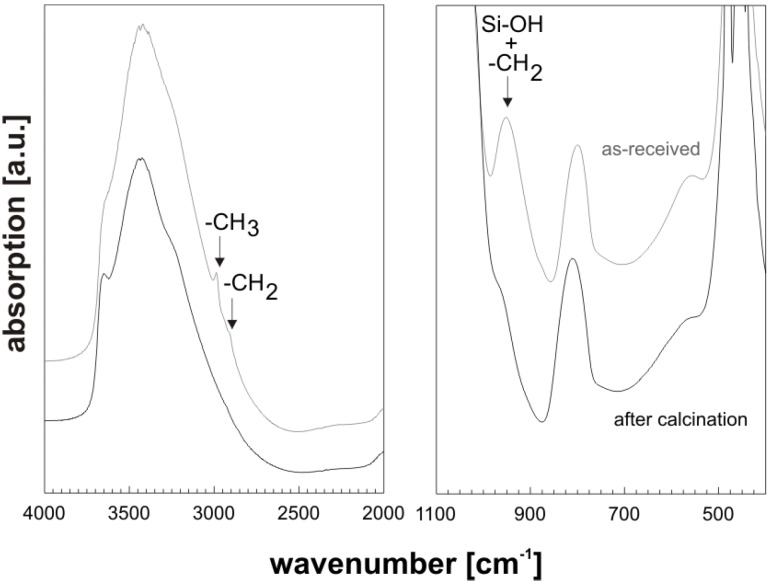
FTIR spectra of commercial silica particles (AngstromSphere™) measured as-received and after calcination at 600 °C in air. Peak positions of organic residues are indicated. The peak around 960 is attributed to surface hydroxyl groups and –CH2 vibrations.

**Figure A3:**
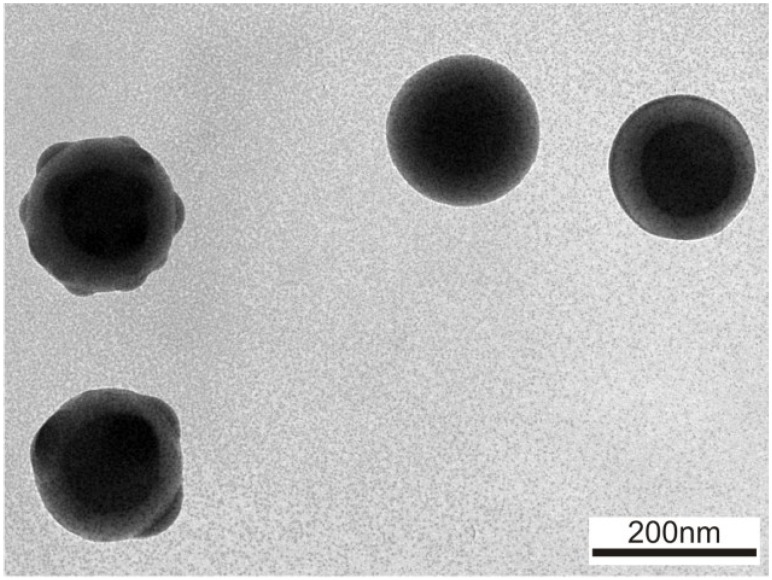
TEM image of commercially available silica particles used without further treatment and coated with molybdenum oxide. The picture shows different morphologies of the coating, likely due to irregular residues on the particle surface, which hinder conformal coating.

As stated above, surface hydroxyl groups favor the decomposition of the precursor on the particle surface (as opposed to homogeneous decomposition in the gas phase) and thus favor a good quality coating. The aerosol based integrated approach is advantageous in this regard because substrate surfaces are very clean and free of residue from solvents, and have a high density of hydroxyl groups [27]. Substrate particles can thus be coated directly in a continuous process to achieve conformal shells. By contrast, experiments in the same coating reactor with as-received commercial grade silica particles did not result in conformal coatings as shown in Figure 3a. This can be attributed to organic residue, which was found in FTIR investigations (see Figure A2) and most likely originated from the liquid based synthesis process of those particles. The residues were apparently not distributed uniformly across the particle surface, leading to variable layer morphologies ranging from completely uncoated to partially or even completely coated surfaces in the same batch (see Figure A3 for further TEM images). When removing this residue by calcination at 600 °C in air for 5 h, it was again possible to obtain conformal coatings with film growth rates similar to the CVS generated particles, as shown in Figure 2 and Figure 3b. Note that the residence times were shorter in this case due to practical limitations with the aerosolization of commercial silica powder, which was done in a fluidized bed.

Similar film growth rates were also obtained for consecutive coating steps to produce core-shell structures with an inner bismuth oxide shell and an outer molybdenum oxide shell as shown in Figure 3c. It was not possible to vary the coating temperature systematically in order to obtain a combined activation energy because of insufficient contrast between layers in the TEM images. However one may assume that the overall activation energy is similar to the values reported above, since it is mostly governed by chemisorption and decarbonylation on already formed molybdenum oxide, except for the first monolayer. Based on an ionic radius of 0.96 Angstrom for Bi^3+^ [26] in the bismuth oxide layer and comparable substrate field strengths given by Loh *et al.* [22], the activation energy for that first molybdenum monolayer on the bismuth oxide shell can be estimated to 102 kJ/mol. This remains to be verified. The film growth of bismuth oxide on silica will be discussed separately in the next paragraph.

### 2.2. Bismuth Oxide Coatings

**Figure 4:**
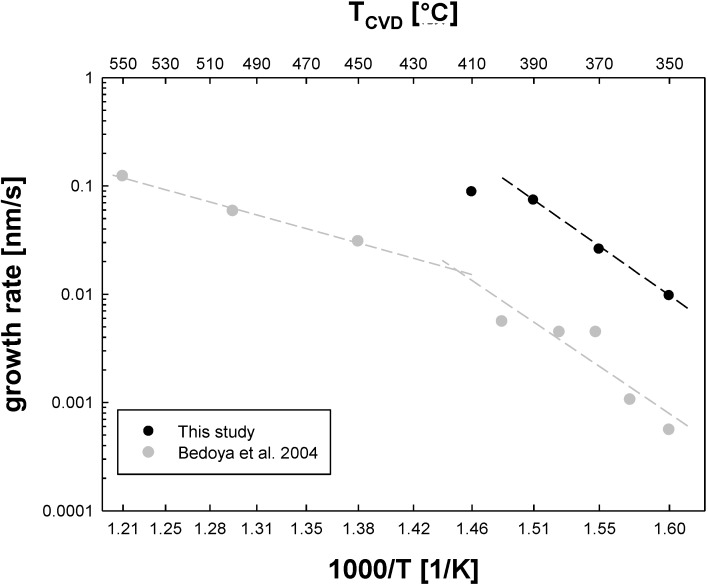
Arrhenius plot for CVD based growth rates of bismuth oxide films on silica particles generated freshly by CVS (black dots) and growth rates derived from Bedoya *et al.* [14] (grey dots). The data of Bedoya *et al.* were fitted with separate regression lines for the temperature ranges with and without significant homogeneous precursor decomposition.

The precursor triphenyl-bismuth requires considerably higher CVD temperatures. Conformal coatings with a closed bismuth oxide shell were obtained in the range of 350–410 °C, as shown in Figure 1. Homogeneous decomposition sets in over about 410 °C. Figure 4 shows the Arrhenius plot for silica particles with a conformal bismuth oxide shell as shown in Figure 1a,b. The activation energy between 350 and 390 °C is 155 ± 10 kJ/mol. One can compare this value to data by Bedoya *et al.* [14], who used the same precursor in a cold wall CVD reactor to coat planar Pt and Ir substrates at reduced pressure. These authors did not take into account the homogeneous decomposition of precursor, which lowers growth rates significantly toward higher temperatures on as shown in Figure 4. While the average activation energy given by the authors was 100 ± 10 kJ/mol, the value for the temperature region of pure film growth (dashed line between 350 and 400 °C) agrees well with our result, despite significant differences in material system, measurement techniques and absolute film thickness.

Following the same line of thought as before, one may assume that precursor deposition now occurs via adsorption and interaction with surface hydroxyl groups by phenyl-ligand substitution, followed by successive complete loss of the ligands. Due to weakening of the metal-phenyl bond by attractive surface groups, the activation energy is somewhat lower than the average bond dissociation energy of 194 ± 10 kJ/mol for the Bi-Ph bond in gas phase triphenyl bismuth as reported by Steele [28].

The growth mechanism can now be described with a Langmuir-Hinshelwood model [14]. Deposition proceeds through simultaneous adsorption of precursor and oxygen molecules on adjacent surface sites, followed by their interaction. The growth rate is thus a function of precursor partial pressure p_Bi_ as given by Equation (1) for a constant oxygen partial pressure [14]:

(1) $$\text{growth rate γ}=\frac{K_{1}∙p_{\text{Bi}}}{{\left(1+K_{2}∙p_{\text{Bi}}\right)}^{2}}$$

with *K*_1_ = 5.51 × 10^−3^ nm/(Pa·s) and *K*_2_ = 9.49 × 10^−3^ Pa^−1^ obtained as fitting parameters to our data.

**Figure 5:**
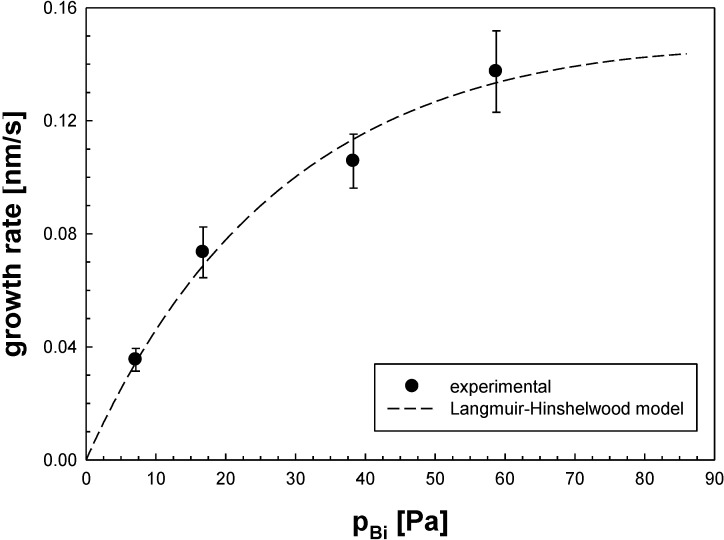
CVD based film growth rates *vs.* partial pressure of the bismuth precursor. Data for bismuth oxide on silica particles generated freshly by CVS, fit function for Langmuir-Hinshelwood growth kinetics.

Figure 5 shows that the film growth rates in our aerosol process are described well by this model. They increase with increasing *p*_Bi_ partial pressure and gradually level off at high values due to the onset of homogeneous gas phase decomposition.

## 3. Experimental Section

Details of the synthesis process can also be found in previous publications [21,29,30]. Silica core particles were synthesized by decomposition of tetra-ethyl-ortho-silicate (TEOS) at 1000 °C in a flow reactor, transferred and sintered to spherical shape at 1500 °C in a second hot wall reactor, and then transferred directly to a coating reactor, to form shells of molybdenum or bismuth oxide films by CVD. All process steps occur in the gas phase at atmospheric pressure. In case of a dual coating, successive coating reactors at 390 °C (bismuth-CVD) and 130 °C (molybdenum-CVD) were applied. The respective precursors for molybdenum oxide and bismuth oxide were molybdenum hexacarbonyl and triphenyl bismuth. A small amount (~10%) of titanium precursor (titanium tetra-isopropoxide) was added in the CVS step to ensure complete sintering to spherical particles. For characterization and determination of the growth rates, particles were directly deposited from the gas phase onto TEM grids by impaction and analyzed without further sample treatment. A Philips CM 12 (120 keV) was used to record the TEM images. The film thickness was determined manually using the ImageJ^©^ software (National Institute of Mental Health, Bethesda, MD, USA). Growth rates were derived based on the calculated residence time in the coating reactor.

Commercial grade 200-nm silica particles (AngstromSphere™, Fiber Optic Center Inc., New Bedford, MA, USA) were aerosolized in a fluidized bed as described by Clemente *et al.* [31] and then fed into the CVD coating reactor. Calcination prior to the experiments was done in air for 5 h at 600 °C. FTIR spectra of the particles were recorded using the postassium bromide disc method and a Vector 22 FTIR spectrometer (Bruker, Ettlingen, Germany).

## 4. Conclusions

An aerosol process combining chemical vapor synthesis and chemical vapor deposition at atmospheric pressure was used to synthesize spherical core-shell structures of silica of 60–120 nm covered with thin layers of molybdenum and/or bismuth oxide in the range of 1–10 nm. Dual layer structures with molybdenum oxide on bismuth oxide were synthesized by two coating reactors in series. The CVS/CVD process is continuous and solvent-free, leading to clean, hydroxylated substrate surfaces and conformal coatings. Commercial grade silica particles coated by the same method required an additional calcination to remove organic residues from the liquid-phase synthesis process which hinder the formation of conformal shells.

Activation energies for film growth from the respective precursors (molybdenum hexacarbonyl and triphenyl bismuth) were found to be 123 ± 10 and 155 ± 10 kJ/mol. These values compare well with data from the literature, provided homogeneous decomposition of the precursor at elevated process temperatures is taken into account. The deposition kinetics for bismuth oxide are described well by a Langmuir-Hinshelwood mechanism.
